# Pharyngolaryngeal Abnormalities viewed via nasoendoscopy associated with Oropharyngeal Dysphagia in Adults: A Scoping Review

**DOI:** 10.1007/s00455-025-10884-6

**Published:** 2025-09-22

**Authors:** Sarah Boggiano, Amy Freeman-Sanderson, Anna Miles, Emma Power, Kris Rogers, Sarah Wallace

**Affiliations:** 1 Discipline of Speech Pathology, Graduate School of Health, Faculty of Health, University of Technology Sydney, PO Box 123 Broadway, Sydney, NSW Australia; 2 Speech Pathology Department, Royal North Shore Hospital, Sydney, Australia; 3 Speech Pathology Department & Intensive Care Unit, Royal Prince Alfred Hospital, Sydney, NSW Australia; 4 Critical Care Division, The George Institute for Global Health, Sydney, NSW Australia; 5 Australian and New Zealand Intensive Care Research Centre (ANZIC-RC), School of Public Health and Preventive Medicine, Monash University, Melbourne, VIC Australia; 6 Speech Science, The University of Auckland, Auckland, New Zealand; 7 Graduate School of Health, Faculty of Health, University of Technology Sydney, Sydney, Australia; 8 The George Institute for Global Health, Sydney, NSW Australia; 9 Manchester University NHS Foundation Trust, Manchester, UK; 10 Division of Infection Immunity and Respiratory Medicine, School of Biological Sciences, Faculty of Biology, Medicine and Health, The University of Manchester, Manchester, UK

**Keywords:** Dysphagia, Swallowing – deglutition, Deglutition disorders, Nasoendoscopy, FEES, Pharynx – larynx

## Abstract

To identify in the literature which pharyngolaryngeal abnormalities (PLA) co-occur or are associated with oropharyngeal dysphagia when viewed via nasoendoscopy. Flexible Endoscopic Evaluation of Swallowing (FEES) is an examination used to assess swallowing. Currently there are no standardized frameworks to observe and report on PLAs observed during a FEES procedure to support diagnostics and inform management. Multiple databases (Scopus (Elsevier), Medline (Ovid), CINAHL (EBSCO) and EMBASE (Ovid)) were searched against inclusion criteria from 1980 to 2024. Key search terms included variations of larynx, pharynx, FEES, and oropharyngeal dysphagia. The study utilized PRISMA-ScR reporting items. Two independent reviewers screened in two phases. Reporting of PLAs with oropharyngeal dysphagia was represented using frequency of co-occurrence and, where available, any statistical analyses attempting to demonstrate an association. Prevalence was calculated for PLA and presence of signs/symptoms of oropharyngeal dysphagia. 117 articles were included for full text review. Data were synthesized into 24 PLA within six categories. PLAs with both frequency of co-occurrence and statistical analysis attempting to demonstrate an association with oropharyngeal dysphagia included unilateral vocal fold motion impairment (UVFMI), velopharyngeal insufficiency, arytenoid motion impairment, incomplete glottic closure, vocal fold atrophy/bowing, edema, and hematoma. This scoping review presents evidence relating to PLAs seen via nasoendoscopy and their reported co-occurrence with oropharyngeal dysphagia. Overall, seven PLA were shown to have an association with oropharyngeal dysphagia, and a further 11 PLA had frequency of co-occurrence with oropharyngeal dysphagia without statistical analysis to support association. Whilst these findings suggest a relationship between PLA and oropharyngeal dysphagia, further research is required to confirm causation of each PLA on swallowing function. Systematic swallowing assessment and use of outcome measures that consider the presence of pharyngolaryngeal abnormalities, will help generate rigorous evidence that is needed to advance precision in diagnostics of swallowing impairment and subsequent interventions.

**Supplementary Information:**

The online version contains supplementary material available at 10.1007/s00455-025-10884-6.

## Introduction

Flexible Endoscopic Evaluation of Swallowing (FEES) is used to assess oropharyngeal swallowing, which is known to be associated with negative consequences, such as increased risks of aspiration, malnutrition, pneumonia and mortality [1, 2]. Observations of swallowing and the pharyngolaryngeal structures during FEES, alongside the ability to determine the consequences of impairments, are essential skills for clinicians. Many scales have been developed to describe swallowing safety and efficiency during FEES, including the New Zealand Secretion Scale [3], Yale Residue Severity Rating Scale [4], and Penetration-Aspiration Scale [5]. These tools use ordinal ratings, consider some element of severity, and correlate well with each other. Identification of accumulated secretions, pharyngeal residue and aspiration are important for establishing an individual’s oropharyngeal dysphagia severity and risk of adverse complications such as malnutrition and pneumonia. However, they do not provide information about the underlying biomechanical cause to guide management decisions. The original FEES examination protocol by Langmore [6] starts with an examination of ‘anatomical-physiological components’ including the appearance of the pharynx and larynx at rest and during movement in functional tasks. This ‘pre-oral trials’ section of the FEES protocol indicates that these observations have relevance. However, guidance for clinicians on how to interpret their significance is limited. There are currently no standardized frameworks which guide the clinician on which tasks to complete, which pharyngolaryngeal abnormalities (PLAs) to report on and how to describe them consistently, and which PLAs to be most concerned about in terms of their potential to impact swallowing safety and efficiency.

There is expanding evidence across various patient populations that documents PLAs and their sequelae for swallowing dysfunction. For example, the use of artificial airways can result in hypopharyngeal and laryngeal edema [7] and injury to the laryngeal mucosa [8, 9]. This is thought to contribute to oropharyngeal dysphagia and subsequent aspiration through localized sensory and movement impairment. Radiotherapy increases tissue thickness, impacting on pharyngeal space and sensation [10] resulting in residue and aspiration [11]. Additionally, up to 56% of patients with unilateral vocal fold palsy present with airway protection difficulties leading to aspiration [12]. This is a common sequela of various and often multiple etiologies, such as neurological impairment in stroke, intracerebral bleeds, as well as progressive neurological diseases, idiopathic causes [13] and intraoperative injuries [14, 15].

Although several PLAs have been reported individually, to date there have been no attempts to systematically analyze the literature base to amalgamate all known PLAs into one framework with standardized terminology to support clinical observations and reporting. Without a common shared taxonomy and an understanding of all factors contributing to a patient’s oropharyngeal dysphagia, interventions such as compensatory strategies and swallowing rehabilitation [16] may be imprecise and ineffective in targeting the underlying physiological contributions to the oropharyngeal dysphagia.

This work will enhance clinical understanding of the impact of these PLAs on oropharyngeal dysphagia, enabling a multidisciplinary approach to determine the most appropriate treatment pathway to mitigate their presence, and in turn improving safety or efficiency of oral intake. A dysphagia team cannot treat oropharyngeal dysphagia based on the observation of aspiration and/or residue alone. For example, vocal fold motion impairment, weak pharyngeal constriction, post-extubation edema and laryngopharyngeal reflux can all result in symptoms of pharyngeal residue and aspiration. Yet, their treatments require considerably different approaches ranging from pharmaceutical therapies (such as steroids or proton pump inhibitors), vocal fold augmentation, or speech pathology led behavioral swallowing exercises [17–19]. A better understanding of the impact of PLAs on swallowing function would also enable clinicians, patients and their families to have a clearer picture of the likely clinical course of recovery of the oropharyngeal dysphagia.

To effectively manage oropharyngeal dysphagia, diagnostic precision is essential. The aim of this study was to identify in the literature which PLAs co-occur or are associated with oropharyngeal dysphagia when viewed via nasoendoscopy. The overall aim being improved understanding of the implications of these PLAs for oropharyngeal dysphagia.

### Methods

**Fig. 1:**
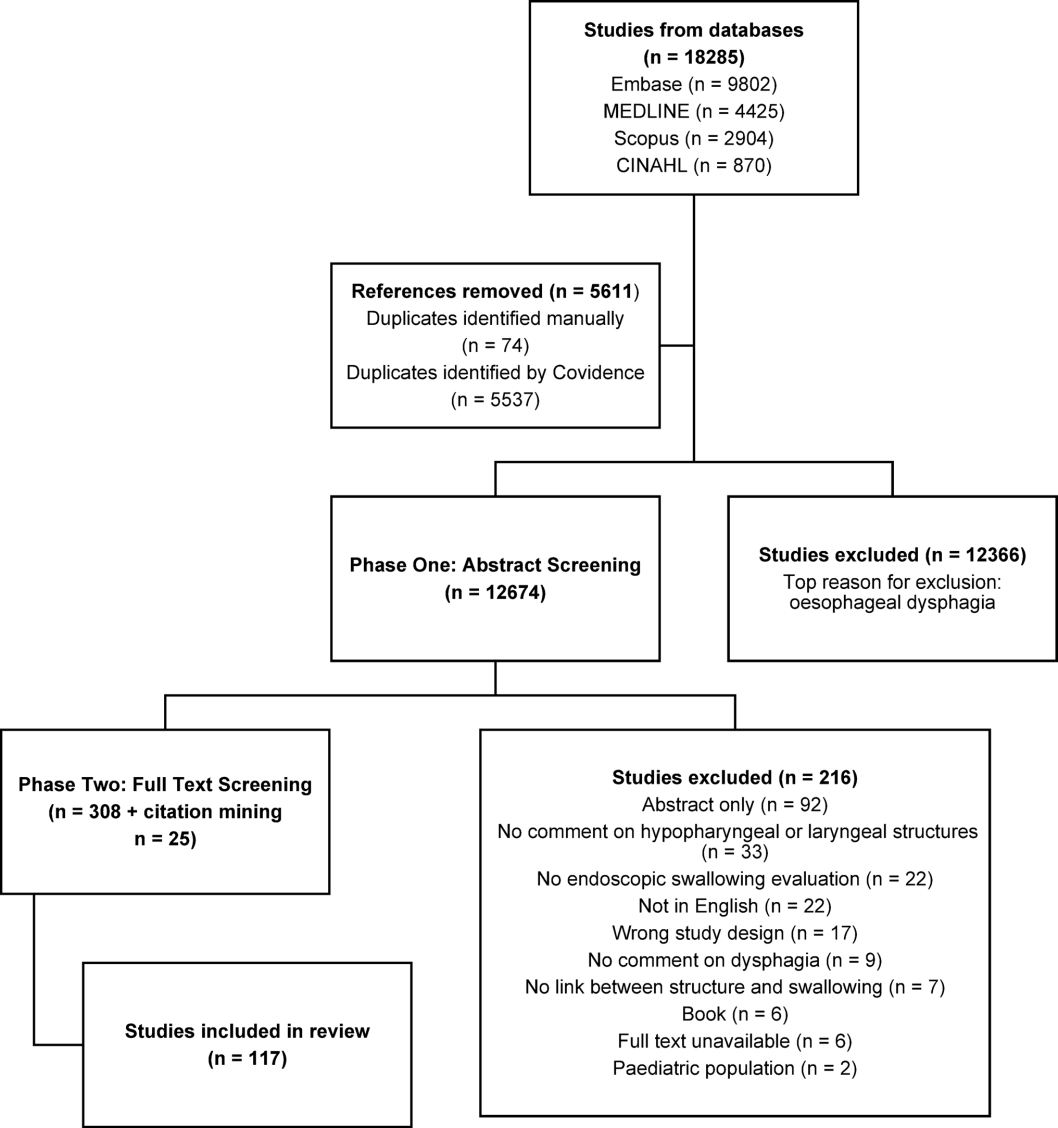
Preferred Reporting Items for Systematic Reviews and Meta-Analyses (PRISMA) Flow Diagram (20/07/2024)

A scoping review was chosen to identify the size and breadth of the developing research evidence [20], to identify gaps in knowledge and summarize the state of the current literature, and to clarify definitions and concepts [21]. A systematic review with critical appraisal was not deemed appropriate as the aim was to provide an overview of the current literature base rather than answer a specific question [21]. Design and reporting were guided by the Preferred Reporting Items for Systematic reviews and Meta-Analyses extension for Scoping Reviews (PRISMA-ScR) [22] checklists. The study protocol was pre-registered on the Open Science Framework (osf.io/3uzr5) (Fig. 1).

### Search Strategy

Detailed search strategy was developed with a librarian (ASH), then agreed by authors (see supporting information). Databases were selected in both medical and allied health fields; Scopus (Elsevier), Medline (Ovid), CINAHL (EBSCO) and EMBASE (Ovid). The search was completed in March 2023 and repeated in July 2024. A date limit from 1980 onwards was applied according to the first published reporting of FEES. Search parameters were refined, with removal of filters for humans and adults (> 18 years) to ensure inclusion of five test articles.

### Study Selection

Any original peer-reviewed research, written in English and reporting on PLAs seen via nasoendoscopy in adults (> 18 years), including swallowing function were included. Further citation mining was completed following full text screening, as one more supplementary search method [23, 24]​. The articles were exported and screened through Covidence (Covidence systematic review software, Veritas Health Innovation, Melbourne, Australia. Available at www.covidence.org).

Screening of records occurred at abstract and full text level. Across both phases, two reviewers screened 25% of the records against the inclusion/exclusion criteria (see Online Resource 1 – Embase Search Strategy). Disagreements were resolved by discussion with the author group. Once the agreement of 0.75 KAPPA, (substantial agreement) [25] was achieved, the first reviewer screened the remainder of the articles in both phases.

### Data Extraction

Following screening, data were extracted from eligible articles by one reviewer using a bespoke excel spreadsheet, developed by the reviewing team. Further data queries were discussed with the author group. Data extracted included study characteristics (year, country, study design, medical/surgical specialty reporting), participant demographics (age, sex), specific PLA, and oropharyngeal dysphagia signs/symptoms reported.

### Data Analysis

Descriptive statistics were used to report demographic data. Content analysis [26] was used to categorize PLAs seen via nasoendoscopy. Data were downloaded, initially the data results were reviewed by SB. The terms were synthesized into subgroups with support from an Ear, Nose and Throat Surgeon (JG) with repeated meetings and discussions with the author team.The category names were refined to ensure they captured all included data. Prevalence data were reported where available. Experimental studies were excluded from prevalence analysis if the participants were recruited for a PLA intervention affecting oropharyngeal dysphagia or airway e.g., vocal fold motion impairment augmentation, as these presented a bias with all participants having an impairment.

Due to limited statistical reporting across articles, we reported attempts to estimate the association between PLAs with oropharyngeal dysphagia, and when available, any statistical analysis used to support this evidence. Meta-analysis was considered where possible to assess the strength of evidence (see below for more detail).

#### Frequency of Reporting of PLAs with Oropharyngeal Dysphagia

Where PLAs and signs/symptoms of oropharyngeal dysphagia were reported, the number of articles and number of participants across articles were tallied to capture the breadth of the literature. Given the unique nature of the data set and no available defined reporting thresholds, frequency of reporting thresholds were determined by the author team a priori. Frequencies were only tallied and reported in detail if there were ≥ 5 articles, or ≥ 50 participants pooled across articles. PLAs reported in fewer than 5 articles or in < 50 participants have been summarized together for the purposes of reporting. Data from all articles are available in Online Resource 5 - Frequency counts of co-occurrence and statistical analysis.

#### Strength of Association between PLAs and Oropharyngeal Dysphagia

Summary study data or risk ratios (RR) were preferentially extracted, followed by p-values then threshold of p-values (e.g. <0.05). Where data were available (> 1 study with statistics completed for a singular PLA), meta-analysis was performed for that combination of PLAs (e.g., unilateral vocal fold motion impairment (UVFMI)) and signs/symptoms of oropharyngeal dysphagia (e.g., aspiration). A random-effects meta-analysis using the restricted maximum likelihood estimator was carried out. Heterogeneity within a pathophysiological group was assessed with I^2^ and τ^2^.

## Results

The search yielded 12,674 articles after de-duplication. 3,108 articles’ titles and abstracts (25%) were screened by both reviewers; 12,366 were excluded. 308 articles advanced to full text screening. Due to heterogeneity of definitions in the articles, 33% (*n* = 101) of abstracts were screened by both reviewers, with a high level of agreement (K = 0.75) [25]. Two members of the reviewing team were listed authors of included articles, for these, the third reviewer (who was not a listed author) completed the screening to reduce bias. Citation mining with full text review identified a further 25 articles, most frequently due to swallowing assessment not being explicitly described in the title or abstract. Following all stages of screening, 117 articles were included in the review.

### Study Characteristics

Reporting of the co-occurrence between PLA and oropharyngeal dysphagia has exponentially increased in the last 15 years (75% of all papers included in this review). Most articles were from Europe (34%) and North America (32%). 95% of primary authors were Ear, Nose and Throat physicians/surgeons and/or Speech Pathologists. Patients were across a range of medical and surgical specialties and the age range was 18–97 years. Details have been reported in Online Resource 4 - Included articles organized alphabetically by author. Study designs were predominantly observational or case studies. Of the articles which included statistical analyses, there were ten cross-sectional, one cohort, two case-control studies, as well as and three case series.

Included articles reported ≥ 1 PLAs co-occurring with oropharyngeal dysphagia. Most articles reported motion impairment (*n* = 78), followed by obstruction to bolus flow or airflow (*n* = 31), mucosal abnormalities (*n* = 29), glottal insufficiency (*n* = 18), change of shape (*n* = 19), and abnormal movement pattern (*n* = 7). Oropharyngeal dysphagia was reported differently across articles using: validated swallowing outcome measures/scoring systems (*n* = 101), validated patient reported symptom tools (*n* = 18), unvalidated measures and/or patient reported symptoms (*n* = 13), sensory tests (including laryngeal adductor reflex testing or flexible endoscopic evaluation of swallowing with sensory testing (FEESST) (*n* = 37) as well as binary classifications of presence of oropharyngeal dysphagia (*n* = 33), secretion accumulation (*n* = 33), pre-swallow pooling (*n* = 18), aspiration (*n* = 94), residue (*n* = 41) and regurgitation (*n* = 4).

### Identification of Pharyngolaryngeal Abnormalities Seen Via Nasoendoscopy

59 different terms were used to label and report endoscopic PLAs impacting swallowing function. These were synthesized into 32 unique terms in six defined subgroups. Subgroup classification was motion impairment, glottal insufficiency, obstruction to bolus flow or airflow, mucosal abnormalities, change of shape, and abnormal movement pattern (see Table 1 and Online Resource 3 – All listed terminology).

**Table 1:** Categorization into subgroups and summary of nasoenndoscopic pharyngolaryngeal abnormalities

Subgroup	Definition	Pharyngolaryngeal abnormalities
Motion impairment	Structures, which on testing in function, did not have full range of motion.	• True/false vocal fold motion impairment - unilateral/bilateral (not in midline position) • Velopharyngeal insufficiency • Arytenoid motion impairment • Arytenoid prolapse/collapse • Subluxation/ankylosis of cricoarytenoid joint • Laterofixation of vocal fold • Reduced pharyngeal wall movement
Glottal insufficiency	PLAs, which despite complete movement of arytenoids, did not enable complete glottic approximation.	• Incomplete glottic closure (unspecified) • Vocal fold atrophy/bowing • Phonatory gap/chink
Obstruction to bolus flow or airflow	PLAs which impacted on obstruction to airflow or obstruction to bolus flow to any degree; airway closure or opening in the larynx or beyond (obstructing the airway) or bolus passage through the pharynx.	• Edema • Bilateral true vocal fold motion impairment (in midline position) • Glottic/airway stenosis/web, tracheal/airway stenosis/narrowing • Cricopharyngeal prominence • Midline protrusion
Mucosal PLAs	PLAs seen at the mucosal level at any location.	• Acute inflammatory conditions: ulcer/ulceration, granulation/granuloma, sloughing, erythema • Chronic inflammatory conditions: cyst, scarring/adhesions • Acute traumatic changes: hematoma, laryngeal lesion
Change of shape	Change of shape or structure, observed at rest, which deviated from ‘normal anatomy’ due to congenital malformation or treatments such as radiotherapy, surgical input or otherwise.	• Upright epiglottis/epiglottic stump • Partial removal or absence of; epiglottis, base of tongue, tonsil, arytenoids, true/false vocal folds, aryepiglottic folds, pyriform sinus, and lateral walls, supraglottis • Supracricoid laryngectomy
Abnormal movement pattern	Movements which were complete, however pattern e.g., rhythmic changes or tonal changes to timing with opening or otherwise observed.	• Tremulous movements of laryngopharynx

### Frequency of co-occurrence with Oropharyngeal Dysphagia and Statistical Analysis by Subgroup

Across the 117 articles included, there was variation in reporting due to available data. 99 articles were reported using frequency of PLAs co-occurring with oropharyngeal dysphagia alone, the remaining 18 articles included frequency of co-occurrence in addition to p- values to analyze the relationships between individual PLA and oropharyngeal dysphagia. Specific data are available in Online Resource 5 - Frequency counts of co-occurrence and statistical analysis.

### Motion Impairment

Motion impairment was reported in 78/117 articles. In the motion impairment subgroup, the reported symptoms of oropharyngeal dysphagia included sensory impairment, secretion accumulation, and/or pre-swallow pooling, and/or aspiration and/or residue.

Three of the eight abnormalities (UVFMI, velopharyngeal insufficiency, arytenoid motion impairment) had statistical analysis completed indicating an association with oropharyngeal dysphagia. In addition to these, two other abnormalities (bilateral vocal fold motion impairment - not in midline); reduced pharyngeal wall movement) also had a frequency count ≥ 5 articles or ≥ 50 participants co-occurring with oropharyngeal dysphagia. Three abnormalities (arytenoid prolapse/collapse; subluxation/ankylosis of cricoarytenoid joint; laterofixation of vocal fold) did not have statistical analysis or required frequency count for reporting.

Specifically, for UVFMI, meta-analysis indicated an association between UVFMI and aspiration (RR 1.49, 95% CI, 1.15–1.92) with low heterogeneity (I2 = 21%) [27–33]. Prevalence data indicated 80% (95% CI 0.65–0.90) of participants with UVFMI had residue [30] and 41% (95% CI, 0.35–0.47) had aspiration or a weak cough [27–33].

### Glottic Insufficiency

Glottic insufficiency was reported in 20/117 articles. Across glottic insufficiency symptoms of oropharyngeal dysphagia include sensory impairment, secretion accumulation, and/or pre-swallow pooling and/or aspiration and/or residue.

Incomplete glottic closure (unspecified) and vocal fold atrophy/bowing both had statistical analysis completed indicating an association with oropharyngeal dysphagia as well as frequency count of co-occurrence ≥ 5 articles or ≥ 50 participants pooled across all the available literature. Phonatory gap/chink did not have statistical analysis or required frequency count for reporting.

Prevalence data demonstrated 84% (95% CI, 0.77–0.90) of participants with incomplete glottic closure had secretion accumulation [34] and 63% (95% CI, 0.38–0.84) were reported to aspirate [27].

### Obstruction to Bolus Flow or Airflow

Obstruction was reported in 31/117 articles with various symptoms of oropharyngeal dysphagia including sensory impairment, secretion accumulation, and/or pre-swallow pooling and/or aspiration and/or residue. No articles commented on severity of obstruction to bolus flow or airflow across reported PLAs.

Edema had statistical analysis and frequency count with association/co-occurrence to oropharyngeal dysphagia. One article reported association between edema of unspecified location (RR 2.46, 95% CI, 1.53–3.96) [27] and arytenoid edema (RR 1.67, 95% CI, 0.13–20.88) [18] and oropharyngeal dysphagia. Bilateral true vocal fold motion impairment (in midline position) had one article reporting no association with aspiration [35], this was the only article to show negative association.

Glottic, subglottic, and infraglottic airway obstruction only had required frequency count, and the remaining two in this subgroup (cricopharyngeal prominence; midline protrusion) had neither statistical analysis nor frequency count.

Prevalence data indicated 45% (95% CI, 0.35–0.54) of participants with edema - unspecified location [27], 32% (95% CI, 0.17–0.51/0.16–0.50) of participants with arytenoid/interarytenoid edema [36], 36% (95% CI, 0.18-0.0.57) of participants with true vocal fold edema [36] and 20% (95% CI, 0.01–0.72) of participants with subglottic edema/stenosis [36] had reported aspiration. There was also prevalence of 85% (95% CI, 0.72–0.93) interarytenoid +/- arytenoid edema [18] with sensory impairment.

### Mucosal Abnormalities

Mucosal abnormalities were reported in 29/117 articles with all reporting symptoms of oropharyngeal dysphagia including sensory impairment, secretion accumulation, and/or pre-swallow pooling and/or aspiration and/or residue.

Of the seven PLAs in this subgroup, only hematoma had one article reporting an association with sensory impairment [37]. Erythema and ulceration/granulation/granuloma had no statistical analysis completed; however, they did have the required frequency count for reporting. There was evidence from single articles, with rate ratio estimated by our team from summary statistics, for association of vocal fold erythema (RR 1.61, 95% CI, 0.26–10.06) [31] and vocal process granuloma (RR 1.11, 95% CI, 0.38–3.19) [31] with aspiration.

The remaining four PLAs in this subgroup (sloughing; cyst; scarring/synechiae/adhesions; laryngeal lesion) did not have statistical analysis or required frequency count for reporting.

Individual prevalence data indicated 32% (95% CI, 0.16–0.52) risk of aspiration for both arytenoid and vocal fold erythema [36]. Also, a 9% (95% CI, 0.00-0.41) and 32% (95% CI, 0.13–0.57) respective risk of aspiration with vocal process ulceration and granuloma [36].

### Change of Shape

Change of shape was reported in 19/117 articles, none of which included statistical analysis to demonstrate association with oropharyngeal dysphagia.

Frequency of co-occurrence in change of shape has been reported for; partial/complete removal of; epiglottis, base of tongue, true vocal folds, arytenoids and false vocal folds resulting pre swallow pooling and/or secretion accumulation and/or aspiration and/or residue.

PLAs without required frequency count included partial removal or absence of arytenoids; aryepiglottic folds; pyriform sinus and lateral wall; supraglottis, as well as upright epiglottic/epiglottis stump and supracricoid laryngectomy.

### Abnormal Movement Pattern

Abnormal movement patterns were reported in 7/117 articles across the pharyngolarynx. There were no articles with statistical analysis and ≤ 5 articles or ≤ 50 participants across articles for tremulous movements of the laryngopharynx to indicate an association with oropharyngeal dysphagia.

### Mixed Pharyngo-Laryngeal Abnormalities

One article reported a statistical association between grouped laryngeal abnormalities (edema, ulceration, prolapse and unilateral vocal fold motion impairment) and aspiration [38]. Whilst another article reported one or more abnormal laryngeal findings (vocal fold edema, mucosal changes, subglottic stenosis, or motion impairment) were not associated with aspiration [1] (Table 2).

**Table 2:** Clinician’s Guide: Strength of evidence by subgroup

Subgroup	Pharyngolaryngeal abnormalities		Statistical analysis with association to dysphagia	Frequency of co-occurrence (≥ 5 articles or ≥ 50 participants)	Prevalence (%)	Signs/symptoms with statistical support
Motion impairment	Unilateral vocal fold motion impairment [27–33]		Yes	Yes	41% aspiration or weak cough [27–33]	Aspiration [27–33] Pre swallow pooling, and residue [30] Impaired pharyngeal squeeze maneuver [39]
					80% residue [30]	
	Velopharyngeal insufficiency [31]		Yes	Yes		Aspiration [31]
	Arytenoid motion impairment [27]		Yes	Yes		Aspiration [27]
	True vocal fold motion impairment – bilateral not in midline		No	Yes		
	Reduced pharyngeal wall movement		No	Yes		
	• Arytenoid prolapse/collapse • Subluxation/ankylosis of cricoarytenoid joint • Laterofixation of vocal fold		No			
Glottal insufficiency	Incomplete glottic closure [27, 34]		Yes	Yes	84% secretion accumulation [34]	Secretion accumulation [34] Aspiration [27]
					63% aspiration [27]	
	Vocal fold atrophy/bowing [40, 41]		Yes	Yes		Aspiration [40, 41]
	Phonatory gap/chink		No			
Obstruction to bolus flow or airflow	Edema (unspecified location) [27]		Yes	Yes	45% aspiration [27]	Aspiration [27]
	Edema (Inter/arytenoid) [18]		Yes		85% sensory impairment [18]	Sensory impairment* [18] Residue [42]
	Edema (false vocal folds, interarytenoid, epiglottis, aryepiglottic folds, valleculae, pyriform sinus) [42]		Yes			Residue [42] Sensory impairment (pyriform sinus only) [42]
	Bilateral true vocal fold motion impairment in midline position [35]		Yes	Yes		No association with aspiration [35]
	Glottic/airway stenosis/narrowing		No	Yes		
	Edema (true vocal folds)		No	Yes		
	• Cricopharyngeal prominence • Midline protrusion		No			
Mucosal abnormalities	Hematoma [37]		Yes	Yes		Sensory impairment* [37]
	Ulcer/ulceration, granulation/granuloma		No	Yes	9% vocal process ulcer [36] 32% vocal process granuloma [36]	
	Erythema		No	Yes	31% arytenoid [36] 32% vocal fold [36]	
	• Cyst • Scarring/adhesions	• Laryngeal lesion	No			
Change of shape	Partial removal or absence of tonsil		No	Yes		
	Partial removal or absence of true vocal folds		No	Yes		
	Partial removal or absence of false vocal folds		No	Yes		
	Partial removal or absence of epiglottis		No	Yes		
	Partial removal or absence of base of tongue		No	Yes		
	• Partial removal or absence of; arytenoids, aryepiglottic folds, pyriform sinus, and lateral walls, supraglottis • Upright epiglottis/epiglottic stump • Supracricoid laryngectomy		No			
Abnormal movement pattern	Tremulous movements of laryngopharynx		No			

*Tested with laryngeal adductor reflex testing or flexible endoscopic evaluation of swallowing with sensory testing (FEESST).

## Discussion

This scoping review is the first attempt to summarize the literature reporting PLAs and their co-occurrence with oropharyngeal dysphagia. This contribution contrasts with previous work which focused on specific patient populations e.g., neurological cohorts [43], post intubation [27], functional assessment protocols [6, 44] and specific PLAs in isolation [45]. Determining causation between the PLAs and oropharyngeal dysphagia was not possible based on the current scientific evidence-base. However co-occurrence and association were observed across numerous PLAs. To provide a clinically pertinent summary of available data, we determined a priori to detail results where PLAs were reported in cut-off at least ≥ 5 articles and/or > 50 participants across articles. However, further research is needed to build on our findings to determine the impact of the PLAs in isolation or to better understand their contribution to motoric or sensory deficits in swallowing.

This review found that some PLAs have been reported with statistical analysis, perhaps demonstrating a higher consistency of association, however it should be noted that there was insufficient evidence on the likelihood of presenting with oropharyngeal dysphagia than others. These included UVFMI, velopharyngeal insufficiency, arytenoid motion impairment, incomplete glottic closure, vocal fold atrophy/bowing, edema, and hematoma. Despite the information found in this review of the literature, clinicians should not discount other PLAs being associated with oropharyngeal dysphagia due to heterogenous labelling or variable ease of visibility during FEES, resulting in more frequent acknowledgement and reporting of these PLA.

Whilst research in this field has increased exponentially in the last 15 years; reflected in the 117 articles included in the full text review, the strength of current available evidence associating abnormalities and oropharyngeal dysphagia and the management of these have not been directly addressed in the literature. The purpose of this scoping review was to understand co-occurrences and where possible associations to start building a framework to support clinical diagnosis and management. When there were multiple abnormalities and co-existing neurological changes, it was difficult to understand causation, casual direction, and strength of causation for oropharyngeal dysphagia. One of the primary limitations in the current literature base was that the observation of PLAs was a secondary outcome and not the primary objective of the research. Secondly, only a minority of articles (18/117) utilized statistical analysis of PLAs and their association with oropharyngeal dysphagia and only p-values were utilized. Just four of these articles specifically aimed to assess the impact of multiple abnormalities on oropharyngeal dysphagia [1, 18, 27, 36] and completed statistical analysis, allowing for a direct comparison. And finally, PLAs were often reported concurrently, and it was not possible to distinguish the true impact of each change individually. For these reasons, of the remaining 99 articles, the authors described frequency of co-occurrence of PLAs with oropharyngeal dysphagia.

Another issue, impacting direct comparison, was the lack of a standardized outcome measure for oropharyngeal dysphagia and the vast range of assessment parameters used. Oropharyngeal dysphagia was predominantly rated by health care professionals with a dearth of patient reported symptoms. Many papers reported aspiration as the sole indicator whereas the impact of oropharyngeal dysphagia is much wider, affecting the safety and efficiency of swallowing, resulting in poor outcomes such as malnutrition and dehydration [46]. This heterogeneity of measures may result in under reporting or inaccurate detection of oropharyngeal dysphagia and suggests that any associations between PLAs and oropharyngeal dysphagia symptoms presented in this work should be interpreted cautiously. Future research should consider the use of systematic approaches in FEES to ensure all information is integrated in assessment and reporting, such as the work by Dziewas et al. for patients with neurogenic oropharyngeal dysphagia [47].

Other studies used alternative swallowing investigations e.g., videofluoroscopy (VFSS) or validated outcome measures known to correlate with oropharyngeal dysphagia (e.g., EAT-10 [48], DYMUS [49] DYPARK [50]) alongside functional nasoendoscopy, however these were excluded from our review as they did not use FEES specifically. This data has been excluded from this research and demonstrates a gap where knowledge of PLAs and oropharyngeal dysphagia are known through different means of assessment. Synthesis of these studies may contribute to our understanding of PLAs associated with oropharyngeal dysphagia and should be considered in future work.

The wide breadth of terminology used to describe PLAs indicates a need to develop unambiguous homogenous taxonomy and definitions shared across professions. This would facilitate clinicians and researchers to systematically apply health information and research in a more clinically useful way for their patients [51]. Further refining of these subgroups and terminology into a standardized framework would be valuable for clinicians interpreting FEES images. With further refinement, this could potentially provide clinicians with a summary of the known evidence to be used clinically during FEES, to support the interpretation of the laryngopharyngeal images in clinical decision-making.

Overall, this study highlights the lack of strength of current evidence and the heterogeneous nature of reporting of oropharyngeal dysphagia and the terminology used to describe PLAs. Although many of the abnormalities do not have unequivocal evidence demonstrated through co-occurrence, and causation is not possible to prove currently, clinicians should not disregard the PLAs but be aware of the gaps in research.

### Limitations

Some limitations exist in this scoping review. Whilst the authors attempted to ensure maximal inclusion of all relevant articles, this review was limited to English and despite considerable search terms, the vast array of terminology in the articles could have resulted in omitting articles. Frequency of reporting thresholds were selected as a team and although this allowed us to represent and interrogate the results of the published data, they may not have adequately represented the frequency and impact of all PLA. Design limitations among the included studies prevented comparisons being drawn particularly when PLAs was co-occurring and reported concurrently. Causation is not possible to prove based on the studies available.

## Conclusions

Pharyngolaryngeal abnormalities are observed during FEES and their impact on swallowing function should be considered and integrated into reasoning when assessing sensory and motoric function. This scoping review systematically summarized the available research on PLAs viewed via nasoendoscopy and their association or frequency of co-occurrence with oropharyngeal dysphagia.

Clinicians should be aware of the current evidence base, however more systematic, high-quality research, utilizing consistent terminology for the PLA and outcome measures are required to improve our understanding of direction and strength of causation of PLA in oropharyngeal dysphagia.

Development of an evidence-based framework for clinicians is required, with the aim of improving accuracy of observations and reporting of PLA. Despite the challenges in the current evidence-base for determining the impact of PLA on swallowing function, continued work in this area is critical to provide a complete view of swallowing function to provide optimal and evidence-based care for our patients.

## Supplementary Information

Below is the link to the electronic supplementary material.

Supplementary Material 1

Supplementary Material 2

Supplementary Material 3

Supplementary Material 4

Supplementary Material 5

## Data Availability

The authors confirm that the data supporting the findings of this study are available within the article and its supplementary materials.
