# Supplementary material for: Pharyngolaryngeal Abnormalities viewed via nasoendoscopy associated with Oropharyngeal Dysphagia in Adults: A Scoping Review
Source: Dysphagia. 2025 Sep 22;41(2):358–69. doi: 10.1007/s00455-025-10884-6 (PMC13099671; doi:10.1007/s00455-025-10884-6)
Supplement: Supplementary file 1 — Supplementary Material 1 [file 455_2025_10884_MOESM1_ESM.pdf]

Online Resource *1: Embase (Ovid) Search Strategy*

1	endos*.ti,ab.	471170
2	"Videolaryngoscop* ".ab,ti.	2241
3	"Videoendoscop* ".ab,ti.	1332
4	"Flexible Endoscopic Evaluation of Swallowing".ab,ti.	286
5	"Fibreoptic Endoscopic Evaluation of Swallowing".ab,ti.	79
6	FEES.ab,ti.	12811
7	"Nasendoscop* ".ab,ti.	601
8	"Laryngoscop* ".ab,ti.	22257
9	"Laryngeal video recordings".ab,ti.	4
10	"Flexible Nasendoscopy".ab,ti.	123
11	"Instrumental evaluation".ab,ti.	682
12	"Laryngologist Evaluation".ab,ti.	1
13	1 or 2 or 3 or 4 or 5 or 6 or 7 or 8 or 9 or 10 or 11 or 12	505484
14	Dysphagia.ab,ti.	58038
15	Swallowing.ab,ti.	38582
16	Pharyngeal.ab,ti.	32999
17	Residue.ab,ti.	174194
18	Penetration.ab,ti.	99820
19	Aspiration.ab,ti.	131526
20	Secretions.ab,ti.	38761
21	deglutition.ab,ti.	3060
22	14 or 15 or 16 or 17 or 18 or 19 or 20 or 21	539080
23	13 and 22	38356
24	pyriforms.mp.	27
25	piriforms.mp.	9
26	larynx.mp.	91190
27	"anterior commissure".mp.	3768
28	arytenoid.mp.	2700
29	interarytenoid.mp.	347
30	"posterior commissure".mp.	1626
31	"ar?epiglottic fold".mp.	418
32	"laryngeal vestibule".mp.	369
33	"vocal process".mp.	270
34	"ventricular fold".mp.	110
35	"vocal fold".mp.	8383
36	cord.mp.	423651
37	glottic.mp.	7167
38	subglottic.mp.	7171
39	"laryngotracheal stenosis".mp.	901
40	oedema.mp.	43847
41	congestion.mp.	44022
42	erythema.mp.	122253
43	inflammation.mp.	1155734
44	ulceration.mp.	45911
45	granulation.mp.	34460
46	"mucosal trauma".mp.	233
47	paresis.mp.	33922
48	palsy.mp.	103060
49	subluxation.mp.	16171
50	ankylosis.mp.	7556
51	pachydermia.mp.	212
52	laryngitis.mp.	5030
53	"upper airway".mp.	23833

54	throat.mp.	71240
55	pharynx.mp.	47824
56	laryngopharynx.mp.	554
57	"pharyngeal wall".mp.	2039
58	"base of tongue".mp.	2093
59	epiglottis.mp.	5974
60	valleculae.mp.	270
61	"pre-epiglottic space".mp.	108
62	"lateral channels".mp.	57
63	24 or 25 or 26 or 27 or 28 or 29 or 30 or 31 or 32 or 33 or 34 or 35 or 36 or 37 or 38 or 39 or 40 or 41 or 42 or 43 or 44 or 45 or 46 or 47 or 48 or 49 or 50 or 51 or 52 or 53 or 54 or 55 or 56 or 57 or 58 or 59 or 60 or 61 or 62	2139677
64	23 and 63	8965
