# Supplementary material for: Pharyngolaryngeal Abnormalities viewed via nasoendoscopy associated with Oropharyngeal Dysphagia in Adults: A Scoping Review
Source: Dysphagia. 2025 Sep 22;41(2):358–69. doi: 10.1007/s00455-025-10884-6 (PMC13099671; doi:10.1007/s00455-025-10884-6)
Supplement: Supplementary file 2 — Supplementary Material 2 [file 455_2025_10884_MOESM2_ESM.pdf]

Online Resource ***3: All listed terminology in evidence base***

**Grouping for purposes of data extraction**	**Terminology reported across included articles**
**Edema**	Edema (epiglottic, base of tongue, pharyngoepiglottic folds, interarytenoid, arytenoid, anterior commissure, true vocal folds, false vocal folds, aryepiglottic folds, laryngeal laryngotracheal, posterior pharyngeal wall, subglottic)<br>Cricopharyngeal prominence<br>Chronic laryngitis
**Mucosal changes**	Erythema (epiglottic, interarytenoid, arytenoid, ventricular fold, vocal fold, laryngeal)<br>Pathological findings<br>Ulcer/ulceration<br>Granulation/granuloma<br>Unspecified lesion<br>Scarring<br>Malignant tumour<br>Trauma<br>Haematoma<br>Leukoplakia<br>Pharyngocele<br>Sloughing<br>Cyst<br>Synechiae<br>Epithelial Hyperplasia<br><u>Locations:</u> laryngotracheal, hypopharyngeal epiglottic, vallecular, interarytenoid, arytenoid, vocal fold, aryepiglottic fold, vocal process, supraglottic, subglottic)
**Structural/ movement**	Midline protrusion<br>Protrusion<br>Velopharyngeal insufficiency<br>Upright epiglottis<br>Epiglottic stump<br>Arytenoid prolapse<br>Arytenoid collapse<br>Impaired arytenoid mobility<br>Impaired vocal fold movement<br>Pharyngeal reduced mobility<br>Pharyngeal wall reduced mobility<br>Vocal fold atrophy<br>Pseudosulcus vocalis<br>Unilateral true vocal fold reduced movement (midline, paramedian, abducted, adducted)<br>Unilateral true vocal fold immobility (midline, paramedian, abducted, adducted)<br>Laterofixation<br>Luxation of arytenoid cartilage<br>Ankylosis of cricoarytenoid cartilage<br>Bilateral true vocal fold reduced movement (midline, paramedian, abducted, adducted)<br>Bilateral true vocal fold immobility (midline, paramedian, abducted, adducted)<br>Hyperfunctional dysphonia<br>Incomplete glottic closure<br>Vocal fold bowing<br>Tremulous movement of laryngopharynx<br>Glottic stenosis<br>Glottis web

	Subglottic stenosis<br>Subglottic narrowing<br>Tracheal stenosis<br>Tracheal narrowing<br>Airway stenosis<br>Airway narrowing<br>Impaired upper esophageal opening<br>Cricopharyngeal spasm
**Surgical/anatomical**	Partial removal of structure<br>Absence of structure<br>Surgical flap to structure<br><u>Locations:</u> epiglottis, tonsil, base of tongue, arytenoid, posterior commissure, truce vocal fold, false vocal fold, aryepiglottic folds, pyriform sinus, lateral wall, supraglottic.<br>Partial supracricoid laryngectomy
