# Supplementary material for: Pharyngolaryngeal Abnormalities viewed via nasoendoscopy associated with Oropharyngeal Dysphagia in Adults: A Scoping Review
Source: Dysphagia. 2025 Sep 22;41(2):358–69. doi: 10.1007/s00455-025-10884-6 (PMC13099671; doi:10.1007/s00455-025-10884-6)
Supplement: Supplementary file 3 — Supplementary Material 3 [file 455_2025_10884_MOESM3_ESM.pdf]

Online Resource *4: Included articles organized alphabetically by author*

	**Table 3: Included articles organized alphabetically by author**											
**Authors**	**Title**	**Year**	**Country**	**Study design**	**Patient population**	**Participants who had FEES**	**Motion impairment**	**Glottal insufficiency**	**Obstruction to bolus flow or airflow**	**Mucosal abnormalities**	**Change to shape of structure**	**Abnormal movement pattern**
Alaskarov, E., Batıoğlu-Karaaltın, A., Erdur, Z. B., Gülmez, Z. D., İnan, H. C., Öztürk, Ö	The Long Term Results of Hyaluronic Acid/Dextranomer Injection Laryngoplasty in Unilateral Vocal Fold Paralysis	2024	Turkey	Cohort	Surgical – Laryngology	40	x					
Alfaris AM, Bawazir SM, Al Awaji NN	Retrograde fiberoptic endoscopy evaluation for patients with laryngeal edema.	2020	Saudi Arabia	Cross-Sectional	Medical – Critically ill	36	x		x	x		
Almas, S., Jeffery, C. C.	Late laryngeal dysfunction in head and neck cancer survivors	2023	Canada	Cross-Sectional	Surgical - Head and Neck Oncology	30	x		x			
Almeida VPB, Félix L, Tavares TL, da Silva Castro MM, Tiago RSL.	Dysphagia in patients with coronavirus disease undergoing orotracheal intubation	2022	Brazil	Cross-Sectional	Medical – Critically ill	60	x		x	x		
Alsavaf, M. B., Tardif, J., Salem, E. H., Matrka, L., Carrau, R. L.	A Novel Approach for the Treatment of Intractable Aspiration After Supracricoid Laryngectomy	2023	USA	Case Series	Surgical - Head and Neck Oncology	1					x	
Ambika RS, Datta B, Manjula, BV, Warawantkar, UV & Thomas, AM	Fiberoptic Endoscopic Evaluation of Swallow (FEES) in Intensive Care Unit Patients Post Extubation	2019	India	Cross-Sectional	Medical – Critically ill	41	x		x	x		
Anderson TD, Mirza N.	Immediate percutaneous medialization for acute vocal fold immobility with aspiration	2001	USA	Case series	Mixed	11	x			x		
Arakawa-Sugueno L, Ferraz AR, Morandi J, Capobianco DM, Cernea CR, Sampaio MA, Kulcsar MA, Simões CA, Brandão LG.	Videoendoscopic Evaluation of Swallowing After Thyroidectomy: 7 and 60 Days	2015	Brazil	Case series	Surgical - Laryngology	15	x					
Atkins BZ, Trachtenberg MS, Prince-Petersen R, Vess G, Bush EL, Balsara KR, Lin SS, Davis RD Jr	Assessing oropharyngeal dysphagia after lung transplantation: altered swallowing mechanisms and increased morbidity.	2007	USA	Cross-Sectional	Surgical – Cardiothoracic	131	x					
Aviv JE, Liu H, Parides M, Kaplan ST, Close LG.	Laryngopharyngeal sensory deficits in patients with laryngopharyngeal reflux and dysphagia	2000	USA	Case-Control	Medical – Laryngology	79			x	x		
Aviv JE, Parides M, Fellowes J, Close LG.	Endoscopic evaluation of swallowing as an alternative to 24-hour pH monitoring for diagnosis of extraesophageal reflux	2000	USA	Case-Control	Medical – Laryngology	40			x	x		
Bartholow, A. H.	Fiberoptic endoscopic assessment of dysphagia in a patient with cutaneous and oropharyngeal blisters	2024	USA	Case study	Medical - Dermatology	1				x		

Bartier S, Bodez D, Kharoubi M, Canouï-Poitrine F, Chatelin V, Henrion C, Coste A, Damy T, Béquignon E.	Pharyngo-laryngeal involvement in systemic amyloidosis with cardiac involvement: a prospective observational study	2019	France	Cross-Sectional	Medical – Infectious Diseases	56	x		x	x		
Bekelis K, Gottfried ON, Wolinsky JP, Gokaslan ZL, Omeis I	Severe dysphagia secondary to posterior C1-C3 instrumentation in a patient with atlantoaxial traumatic injury: a case report and review of the literature.	2010	USA	Case report	Surgical - Neurosurgery	1	x					
Benjapornlert P, Kagaya H, Shibata S, Matsuo K, Inamoto Y, Kittipanya-Ngam P, Saitoh E.	The prevalence and findings of fibre-optic endoscopic evaluation of swallowing in hospitalised patients with dysphagia	2020	Japan	Cohort	Mixed	609		x				
Best SRA, Ahn J, Dhillon V, Akst, LM, Hillel AT & Blakeley J	Voice and Swallowing Dysfunction in Neurofibromatosis 2	2016	USA	Cross-Sectional	Medical - Neurological	31	x					
Blumin JH, Pcolinsky DE, Atkins JP	Laryngeal findings in advanced Parkinson's disease.	2004	USA	Cross-Sectional	Medical - Neurology	15	x	x				x
Boggiano S, Williams T, Gill SE, Alexander PD, Khwaja S, Wallace S, McGrath BA	Multidisciplinary management of laryngeal pathology identified in patients with COVID-19 following trans-laryngeal intubation and tracheostomy	2022	Australia	Cross-Sectional	Medical – critically ill	16	x	x	x	x		
Butler SG, Postma GN, Halum SL.	Dysphagia following anterior cervical fusion	2005	USA	Case report	Surgical - Neurosurgery	1				x		
Caliceti U, Tesei F, Scaramuzzino G, Sciarretta V, Brusori S, Ceroni AR.	Videofluoroscopy and videoendoscopy in evaluation of swallowing function in 31 patients submitted to surgery for advanced buccopharyngeal carcinoma.	2004	Italy	Case series	Surgical - Head and Neck Oncology	5					x	
Carrau RL, Pou A, Eibling DE, Murry T, Ferguson BJ.	Laryngeal framework surgery for the management of aspiration	1999	USA	Case series	Surgical – Laryngology	25	x	x				
Castellano, DM, Sinacori, JT & Karakla, DW	Stridor and dysphagia in diffuse idiopathic skeletal hyperostosis (DISH)	2006	USA	Case report	Surgical – Laryngology	1			x			
Charters EK, Bogaardt H, Freeman-Sanderson AL, Ballard KJ, Davies S, Oates J, Clark J	Early fiberoptic endoscopic evaluation of swallow in transoral robotic surgery: Description of swallow function and recovery in the acute postoperative period for oropharyngeal squamous cell carcinoma	2021	Australia	Case series	Surgical - Head and Neck Oncology	21		x	x		x	
Chiba, Y, Sano, D, Ikui, Y, Nishimura, G, Yabuki, K, Arai, Y, Tanabe, T, Ikemiyagi, H, Hyakusoku, H & Oridate, N	Predictive value of the Hyodo score in endoscopic evaluation of aspiration during swallowing	2018	Japan	Cross-Sectional	Mixed	528	x					
Chou, FF., Chen, JB, Huang, SC., Chan, YC, Chi, SY, Lai, CC, Wu, YJ & Chang, HC	Changes in voice quality, swallowing, and pulmonary function after parathyroidectomy for secondary hyperparathyroidism	2022	Taiwan	Case-Control	Surgical – Laryngology	38		x				
Cristalli G, Ferri E, Di Maio P, Spriano G, Mercante G, Ferreli F, Pellini R, Boscolo Nata F	Lateral conservative approach for recurrent/persistent hypopharyngeal carcinoma: a case series.	2020	Italy	Case series	Surgical - Head and Neck Oncology	3					x	
Csanády M, Czigner J, Vass G, Jóri J	Transoral CO2 laser management for selected supraglottic tumors and neck dissection.	2011	Hungary	Case series	Surgical - Head and Neck Oncology	8					x	

Doruk, C., Çaytemel, B., Şahin, E., Kara, H., Samancı, B., Abay, S. N., Bilgiç, B., Hanağası, H., Başaran, B., Enver, N., Rameau, A.	Evaluation of Post-Swallow Residue with Visual Analysis of Swallowing Efficiency and Safety in Patients with Idiopathic Parkinson's Disease	2023	Turkey	Cross-Sectional	Medical - Neurological	53		x				
Dotevall, H., Tuomi, L., Lindell, E., Finizia, C.	Long-term effects on swallowing and laryngeal function after treatment for severe COVID-19 disease in intensive care	2024	Sweden	Cross-Sectional	Medical – Critically ill	36	x			x		
Ebisumoto, K., Sakai, A., Iijima, H., Goto, F., Yamauchi, M., Maki, D., Teramura, T., Wasano, K., Okami, K.	Swallowing Function and Quality of Life in Patients Treated With Transoral Videolaryngoscopic Surgery for Pharyngolaryngeal Cancer	2024	Japan	Cross-Sectional	Surgical - Head and Neck Oncology	73						x
Eckel HE, Thumfart M, Wassermann K, Vössing M, Thumfart WF	Cordectomy versus arytenoidectomy in the management of bilateral vocal cord paralysis.	1994	Germany	RCT	Surgical - Head and Neck Oncology	36					x	
El Fassi N, Gallois Y, Crestani S, Fichaux-Bourrin P, Ory F, Fabbri M, Pavy le Traon A, Woisard V.	Pharyngolaryngeal semiology and prognostic factors in multiple system atrophy.	2022	France	Cross-Sectional	Medical – Neurological	84	x	x				x
Enver N, Borders JC, Curtis JA, Sevitz JS, Vanegas-Arroyave N, Troche MS	The Role of Vocal Fold Bowing on Cough and Swallowing Dysfunction in Progressive Supranuclear Palsy.	2021	USA	Cross-Sectional	Medical - Neurological	23		x				
Erwood MS, Walters BC, Connolly TM, Gordon AS, Carroll WR, Agee BS, Carn BR, Hadley MN.	Voice and swallowing outcomes following reoperative anterior cervical discectomy and fusion with a 2-team surgical approach.	2018	USA	Case series	Surgical - Neurosurgery	67	x					
Fang TJ, Li HY, Tsai FC, Chen IH	The role of glottal gap in predicting aspiration in patients with unilateral vocal paralysis.	2004	Taiwan	Cross-Sectional	Mixed	36	x					
Fourdrain A, De Dominicis F, Iquille J, Lafitte S, Merlusca G, Witte Pfister A, Bagan P, Berna P.	Usefulness of a routine endoscopic assessment of laryngeal lesions after lung cancer surgery	2018	France	Cross-Sectional	Surgical – cardiothoracic	250	x			x		
Hammer GP, Tomazic PV, Vasicek S, Graupp M, Gugatschka M, Baumann A, Konstantiniuk P, Koter SH.	Carotid endarterectomy significantly improves postoperative laryngeal sensitivity	2016	Germany	Case series	Surgical - Vascular	26	x		x	x		
Hendricker RM, deSilva BW, Forrest LA.	Gore-Tex medialization laryngoplasty for treatment of dysphagia	2010	USA	Case series	Surgical – Laryngology	47	x					
Ito, H., Nagao, A., Maeda, S., Nakahira, M., Hyodo, M.	Clinical Significance of Surgical Intervention to Restore Swallowing Function for Sustained Severe Dysphagia	2023	Japan	Case series	Surgical – Laryngology	25	x					
Jamal N, Erman A, Chhetri DK.	Partial Epiglottoplasty for Pharyngeal Dysphagia due to Cervical Spine Pathology.	2015	USA	Case series	Surgical – Laryngology	1			x		x	
Jamroz, B., Sobol, M., Chmielewska-Walczak, J., Milewska, M., Niemczyk, K.	The risk factors for silent aspiration: A retrospective case series and literature review	2024	Poland	Case series	Mixed	455	x					
Jensen K, Lambertsen K, Torkov P, Dahl M, Jensen AB, Grau C	Patient assessed symptoms are poor predictors of objective findings. Results from a cross-sectional study in patients treated with radiotherapy for pharyngeal cancer	2007	Denmark	Cross-Sectional	Surgical - Head and Neck Oncology	35	x	x				

Kagaya H, Okada S, Shigeta R, Ogata N, Ota K, Shibata S, Saitoh E.	Dysphagia associated with unilateral vocal cord immobility after cardiovascular surgery.	2011	Japan	Cross-Sectional	Surgical – Cardiothoracic	69	x					
Kammer RE, Jones CA, Johnson AM, Dailey SH, McCulloch TM, Thibeault SL	High-resolution manometry and swallow outcomes after vocal fold injection medialization for unilateral vocal fold paralysis/paresis	2019	USA	Case series	Surgical – Laryngology	17	x					
Karimi E, Hashemi Z, Sohrabpour S, Mousavi SMM, Yousefi J.	Comparison of swallowing disorder in patients with tongue cancer undergoing reconstruction with either a radial forearm free flap or a submental island flap.	2023	Iran	RCT	Surgical - Head and Neck Oncology	26					x	
Kaye GM, Zorowitz RD, BaLowes S	Role of flexible laryngoscopy in evaluating aspiration.	1997	USA	Cross-Sectional	Mixed	105	x	x				
Krasnodębska P, Szkiełkowska A, Jarzyńska-Bućko A, Włodarczyk E, Miaśkiewicz B.	Characteristics of swallowing disorders in patients with dysphonia.	2019	Poland	Cross-Sectional	Mixed	61	x		x			
Krasnodębska P, Jarzyńska-Bućko A, Szkiełkowska A, Miaśkiewicz B, Skarżyński H.	Diagnosis in Muscle Tension Dysphagia	2020	Poland	Cross-Sectional	Medical	61	x					
Krisciunas GP, Langmore SE, Gomez-Taborda S, Fink D, Levitt JE, McKeehan J, McNally E, Scheel R, Rubio AC, Siner JM, Vojnik R, Warner H, White SD, Moss M.	The Association Between Endotracheal Tube Size and Aspiration (During Flexible Endoscopic Evaluation of Swallowing) in Acute Respiratory Failure Survivor	2020	USA	Cross-Sectional	Medical – Critically ill	210				x		
Laccourreye O, Paczona R, Ageel M, Hans S, Brasnu D, Crevier-Buchman L.	Intracordal autologous fat injection for aspiration after recurrent laryngeal nerve paralysis	1999	France	Case series	Surgical – Laryngology	20	x					
Langmore SE, Krisciunas GP, Warner H, White SD, Dvorkin D, Fink D, McNally E, Scheel R, Higgins C, Levitt JE, McKeehan J, Deane S, Siner JM, Vojnik R, Moss M	Abnormalities of Aspiration and Swallowing Function in Survivors of Acute Respiratory Failure	2021	USA	Cross-Sectional	Medical – Critically ill	213	x	x	x	x		
Laskay, N. M. B., Yang, L. C., Estevez-Ordonez, D., Warner, J. D., Trahan, D., Stone, C., Grayson, J. W., Withrow, K., Hadley, M. N.	Early Voice and Swallowing Disturbance Incidence and Risk Factors After Revision Anterior Cervical Discectomy and Fusion Using a Multidisciplinary Surgical Approach: A Retrospective Cohort Evaluation of a Prospective Database	2024	USA	Cross-Sectional	Surgical - Neurosurgery	109	x					
Leder SB, Ross DA	Incidence of vocal fold immobility in patients with dysphagia.	2005	USA	Cross-Sectional	Mixed	1452	x					
Leder SB, Suiter DM, Duffey D, Judson BLV	Vocal fold immobility and aspiration status: a direct replication study.	2012	USA	Case series	Mixed	112	x					
Leder SB, Bayar S, Sasaki CT, Salem RR.	Fiberoptic endoscopic evaluation of swallowing in assessing aspiration after transhiatal esophagectomy	2007	USA	Case series	Surgical – Upper Gastro Intestinal	73	x					

Liu, T., Feng, H., Liang, Z., Xu, S., Qin, G.	Analysis of swallowing and voice-related quality of life in patients after supracricoid partial laryngectomy	2024	China	Cohort	Surgical - Head and Neck Oncology	21					x	
Lu, Y. T., Tseng, W. H., Chiu, H. L., Yang, T. L.	Improvement in swallowing safety after injection laryngoplasty in patients with unilateral vocal paralysis complicated with aspiration	2024	Taiwan	Case series	Surgical – Laryngology	22	x					
Mani, GS, Mathews, S S, Victor, P, Peter, JV, Yadav, B, & Albert, RR	Laryngeal Dysfunction in Acute Organophosphorus and Carbamate Poisoning.	2022	India	Case series	Medical – Critically ill	18	x		x			
Marom T, Flaksman H, Ben-David N, Dabby R, Gilad R, Oestreicher-Kedem Y, Roth Y	Isolated myoclonus of the vocal folds.	2013	Israel	Case series	Medical - Neurological	3						x
Miles, A, McLellan, N, Machan, R, Vokes, D, Hunting, A, McFarlane, M, Holmes, J. & Lynn, K	Dysphagia and laryngeal pathology in post-surgical cardiothoracic patients	2018	New Zealand	Cohort	Surgical – Cardiothoracic	106	x		x	x		
Miles, A, Barua, S, McLellan, N & Brkic, L	Dysphagia and medicine regimes in patients following lung transplant surgery: A retrospective review	2021	New Zealand	Cross-Sectional	Surgical – Cardiothoracic	65	x		x	x		
Miles, A., Hunting, A.	Pharyngeal Squeeze Maneuver During Endoscopy-What Does it Tell Us?	2023	New Zealand	Cross-Sectional	Mixed	222	x					
Mok P, Woo P, Schaefer-Mojica J	Hypopharyngeal pharyngoplasty in the management of pharyngeal paralysis: a new procedure.	2003	USA	Case series	Surgical – Laryngology	7	x					
Mozzanica, F., Pizzorni, N., Eplite, A., Ginocchio, D., Colombo, A., Mora, G., Ambrogi, F., Warnecke, T., Schindler, A.	Swallowing Characteristics in Patients with Multiple System Atrophy Analyzed Using FEES Examination	2024	Italy	Cross-Sectional	Medical - Neurological	25	x	x				
Mueller AH, Hagen R, Foerster G, Grossmann W, Baumbusch K, Pototschnig C	Laryngeal pacing via an implantable stimulator for the rehabilitation of subjects suffering from bilateral vocal fold paralysis: A prospective first-in-human study.	2016	Germany	Case series	Surgical – Laryngology	7	x					
Naunheim M & Langerman A	Pharyngoceles: a photo-anatomic study and novel management.	2013	USA	Case series	Surgical – Laryngology	1				x		
Nawka T, Sittel C, Gugatschka M, Arens C, Lang-Roth R, Wittekindt C, Hagen R, Müller AH, Volk GF, Guntinas-Lichius O.	Permanent transoral surgery of bilateral vocal fold paralysis: a prospective multi-center trial.	2015	Austria	Case series	Surgical – Laryngology	36	x				x	
Nelke C, Labeit B, Meuth SG, Warnecke T, Dziewas R and Ruck T	Bilateral Vocal Fold Paralysis in Myasthenia Gravis: A Case Report and Literature Review.	2020	Germany	Case report	Medical - Neurological	1	x					
NurHashima AR, Marina MB, Sani A.	Cricotracheal separation: a case of delayed diagnosis and treatment.	2011	Malaysia	Case report	Surgical – Laryngology	1	x		x	x		
Oeken J, Hänsch U, Thiel S, Bootz F.	Swallowing function after endoscopic resection of supraglottic carcinoma with the carbon dioxide laser.	2001	Germany	Case series	Surgical - Head and Neck Oncology	14					x	

Ollivere B, Duce K, Rowlands G, Harrison P, O'Reilly BJ.	Swallowing dysfunction in patients with unilateral vocal fold paralysis: aetiology and outcomes.	2006	UK	Cohort	Surgical – Laryngology	15	x					
Órfão, J.. Melo, M.. Soares, M. T., Raposo, D., Alemão, A. R., Barbosa, L., Freire, F.	Swallowing evaluation in patients who underwent Partial CO2 LASER Epiglottectomy for Sleep Apnea treatment	2023	Portugal	Case series	Surgical – Laryngology	8					x	
Ottaviani F, Schindler A, Klinger F, Scarponi L, Succo G, Mozzanica F.	Functional fat injection under local anesthesia to treat severe postsurgical dysphagia, case report.	2019	Italy	Case report	Surgical – Laryngology	1					x	
Paker M, Duek I, Awwad F, Benyamini L, Meshyeev T, Gil Z, Cohen JT.	Long-term swallowing performance following transoral robotic surgery for obstructive sleep apnea	2019	Israel	Cohort	Surgical – Laryngology	14					x	
Pastene, D., Lehrer, E., Jubes, S., Santamaria, J., Iranzo, A., Gaig, C., Vilaseca, I.	Upper airway manifestations of anti-IgLON5 disease: Otorhinolaryngological point of view	2024	Spain	Case series	Surgical – Laryngology	9						
Peretti G, Piazza C, Del Bon F, Mora R, Grazioli P, Barbieri D, Mangili S, Nicolai P.	Function preservation using transoral laser surgery for T2-T3 glottic cancer: oncologic, vocal, and swallowing outcomes.	2013	Germany	Cohort	Surgical - Head and Neck Oncology	Unknown	x				x	
Périé S, Laccourreye O, Bou-Malhab F, Brasnu D.	Aspiration in unilateral recurrent laryngeal nerve paralysis after surgery.	1998	France	Cohort	Surgical – Laryngology	5	x	x				
Périé S, Roubeau B, Lacau St Guily J.	Laryngeal paralysis: distinguishing Xth nerve from recurrent nerve paralysis through videoendoscopic swallowing study (VESS).	2003	France	Cross-Sectional	Mixed	6	x	x				
Pissurno NSCA, Esteves LDM, Benedito JM, Giglio VP, de Carvalho LR, Mendes RP, Paniago AMM	Impact of laryngeal sequelae on voice- and swallowing-related outcomes in paracoccidioidomycosis	2020	Brazil	Cohort	Medical – Infectious Diseases	16				x		
Postma, GN, McGuirt Sr, WF, Butler, SG, Rees, CJ, Crandall, HL & Tansavatdi, K	Laryngopharyngeal Abnormalities in Hospitalized Patients with Dysphagia	2007	USA	Cross-Sectional	Mixed	99	x	x	x	x		
Pou AM, Carrau RL, Eibling DE, Murry T.	Laryngeal framework surgery for the management of aspiration in high vagal lesions.	1998	USA	Case series	Surgical – Laryngology	35	x	x	x			
Printza A, Boziki M, Triaridis S, Kiousi V, Arnaoutoglou M, Constantinidis J, Grigoriadis N.	Tongue strength, dysphagia questionnaire, pharyngeal secretions and FEES findings in dysphagia management in amyotrophic lateral sclerosis	2021	Greece	Cross-Sectional	Medical - Neurological	31	x	x				
Queija DDS, Dedivitis RA, Arakawa-Sugueno L, de Castro MAF, Chamma BM, Kulcsar MAV, de Matos LL.	Cervicofacial and Pharyngolaryngeal Lymphedema and Deglutition After Head and Neck Cancer Treatment.	2019	Brazil	Cross-Sectional	Surgical - Head and Neck Oncology	46			x			
Radhakrishnan S, Menon UK, Anandakuttan A	A combined approach of bedside clinical examination and flexible endoscopic evaluation of swallowing in poststroke dysphagia: A pilot study.	2013	India	Cross-Sectional	Medical - Neurological	16	x					x
Reder, L., Bertelsen, C., Angajala, V., O'Dell, K., Fisher, L.	Hospitalized Patients with New-Onset Vocal Fold Immobility Warrant Inpatient Injection Laryngoplasty	2021	USA	Case Series	Mixed							

Romak JJ, Olsen SM, Koch CA, Ekbom DC.	Bilateral vallecular cysts as a cause of Dysphagia: case report and literature review.	2010	USA	Case report	Surgical – Laryngology	1				x		
Rumbach AF, Ward EC, Cornwell PL, Bassett LV, Muller MJ.	Physiological characteristics of dysphagia following thermal burn injury	2012	Australia	Cross-Sectional	Medical - Burns	19			x	x		
Rumbach AF, Cremer R, Chatwood A, Fink S, Haider S, Yee M.	The Challenges of Dysphagia Management and Rehabilitation in Two Complex Cases Post Chemical Ingestion Injury	2016	Australia	Case report	Medical - Burns	2			x	x		
Sahai, A., Dixit, R., Choudhary, R., Marfatia, H., Mohapatra, P.	Clinical Conundrum: Unveiling a Unique Presentation of Hypopharyngeal Carcinoma	2024	India	Case report	Surgical - Head and Neck Oncology	1	x					
Sandblom H, Dotevall H, Svennerholm K, Tuomi L, Finizia C.	Characterization of dysphagia and laryngeal findings in COVID-19 patients treated in the ICU-An observational clinical study.	2021	Sweden	Cross-Sectional	Medical – Critically ill	25	x		x	x		
Scheel R, Pisegna JM, McNally E, Noordzij JP, Langmore SE.	Endoscopic Assessment of Swallowing After Prolonged Intubation in the ICU Setting.	2016	USA	Cross-Sectional	Medical – Critically ill	59	x		x	x		
Schröder, JB., Melzer, N, Ruck, T, HeidbLower, A, Kleffner, I, Dittrich, R, Muhle, P, Warnecke, T, & Dziewas, R.	Isolated dysphagia as initial sign of anti-IgLON5 syndrome	2017	Germany	Case report	Medical - Neurological	1	x					
Seidler TO, Pèrez Alvarez JC, Wonneberger K, Hacki T.	Dysphagia caused by ventral osteophytes of the cervical spine: clinical and radiographic findings.	2009	Germany	Cross-Sectional	Medical	20			x			
Sellars C, Campbell AM, Stott DJ, Stewart M, Wilson JA.	Swallowing abnormalities after acute stroke: A case control study. Dysphagia.	1999	UK	Case-Control	Medical - Neurological	31	x					
Semmler M, Keck T, Reiter R, Gruen PM.	Endolaryngeal posterior mucosal flap for surgical repair of posterior glottic stenosis.	2011	Germany	Case series	Surgical – Laryngology	4					x	
Sone, M., Mizokami, D., Takihata, S., Shiotani, A., Araki, K.	Characteristic Video Laryngeal Endoscopic "Pharyngeal Rotation" in Unilateral Pharyngeal Constrictor Muscle Paresis: A Case of Herpes Zoster Pharyngitis	2024	Japan	Case report	Medical – Aged care	1	x					
Spiegelberg, M., Ermiş, E., Raabe, A., Tarnutzer, A. A.	Triggered episodic vestibular syndrome and transient loss of consciousness due to a retrostyloidal vagal schwannoma: a case report	2023	Switzerlan d	Case report	Medical - Neurological	1	x					
Stanley C, Paddle P, Griffiths S, Safdar A, Phyland D.	Detecting Aspiration During FEES with Narrow Band Imaging in a Clinical Setting	2022	Australia	Cross-Sectional	Mixed	21	x					
Steinhagen V, Grossmann A, Benecke R, Walter U.	Swallowing disturbance pattern relates to brain lesion location in acute stroke patients.	2009	Germany	Cross-Sectional	Medical - Neurological	60						x
Stomeo F, Rispoli V, Sensi M, Pastore A, Malagutti N, Pelucchi S.	Subtotal arytenoidectomy for the treatment of laryngeal stridor in multiple system atrophy: phonatory and swallowing results.	2016	Brazil	Case report	Surgical - Neurological	1	x				x	
Sun, F., Qiao, J., Huang, X., He, Z., Dou, Z.	Characteristics of post-stroke dysphagia: A retrospective study based on FEES	2023	China	Cross-Sectional	Medical - Neurological	94	x					
Tabaee A, Murry T, Zschommler A, Desloge RB.	Flexible endoscopic evaluation of swallowing with sensory testing in patients with unilateral vocal fold immobility: incidence and pathophysiology of aspiration.	2009	USA	Cross-Sectional	Mixed	81	x		x	x		

Tabaee A, Johnson PE, Gartner CJ, Kalwerisky K, Desloge RB, Stewart MG	Patient-controlled comparison of flexible endoscopic evaluation of swallowing with sensory testing (FEESST) and videofluoroscopy	2006	USA	Cross-Sectional	Not stated	54	x		x	x		
Tai, J., Hu, R., Fan, S., Wu, Y., Wang, T., Wu, J.	Theta-burst transcranial magnetic stimulation for dysphagia patients during recovery stage of stroke: a randomized controlled trial	2023	China	Randomized control trial	Medical - Neurological	45	x					
Tamin S, Adham M, Noer A, Supriana N, Bardosono S.	Upright epiglottis prevents aspiration in patients with nasopharyngeal carcinoma post-chemoradiation.	2021	Indonesia	Cross-Sectional	Surgical - Head and Neck Oncology	39	x		x		x	
Tervonen H, Niemelä M, Lauri ER, Back L, Juvas A, Räsänen P, Roine RP, Sintonen H, Salmi T, Vilkman SE, Aaltonen LM.	Dysphonia and dysphagia after anterior cervical decompression.	2007	Finland	Case-Control	Surgical - Neurosurgery	8	x			x		
Vallamkondu, V, Gatenby, A, Shakeel, M, & Hussain, A.	Mislaid dentures: a cause for unusual presentation of bilateral vocal cord palsy.	2014	UK	Case report	Medical – Aged care	1	x					
Velasco, GC, Artates, A MV, Castañeda, SS & Castillo, RM	Leprosy of the larynx and its clinical manifestations – A case report	2020	Philippines	Case report	Medical – Infectious Diseases	1	x			x	x	
Wolf C, Meiners TH.	Dysphagia in patients with acute cervical spinal cord injury.	2003	Germany	Cross-Sectional	Medical – Spinal cord injury	51	x		x			
Woo P, Kelly G, Kirshner P.	Airway complications in the head injured	1989	USA	Cross-Sectional	Medical - Neurological	50	x		x			x
Yaguchi H, Sakuta K, Mukai T, Miyagawa S.	Fiberoptic laryngoscopic neurological examination of amyotrophic lateral sclerosis patients with bulbar symptoms	2022	Japan	Cross-Sectional	Medical - Neurological	50	x					
Yildiz, M. G.	Vocal Cord Paralysis Following Bee Sting: A Rare Case Report	2023	Turkey	Case report	Medical	1	x					
Yiu Y, Curtis JA, Perry SE, Troche MS.	Relationship of vocal fold atrophy to swallowing safety and cough function in Parkinson's disease.	2020	USA	Cross-Sectional	Medical - Neurological	30		x				x
Yoon JA, Kim SH, Jang MH, Kim SD, Shin YB.	Correlations between Aspiration and Pharyngeal Residue Scale Scores for Fiberoptic Endoscopic Evaluation and Videofluoroscopy	2019	Korea	Cross-Sectional	Mixed	178	x	x				
Yu L, Zheng M, Ren J, Hu J, Lu D, Yang H.	Supracricoid partial laryngectomy with cricohyoidoepiglottopexy for patients with laryngeal cicatricial stenosis: Safety and efficacy.	2021	China	Case series	Surgical – Laryngology	16					x	
Zuniga SA, Ebersole B, Jamal N.	Utility of Eating Assessment Tool-10 in Predicting Aspiration in Patients with Unilateral Vocal Fold Paralysis.	2018	USA	Case series	Mixed	35	x					
Zuniga S, Ebersole B, Jamal N.	Improved swallow outcomes after injection laryngoplasty in unilateral vocal fold immobility.	2018	USA	Case series	Surgical – Laryngology	21	x					

Zuniga S, Ebersole B, Jamal N.	Inpatient injection laryngoplasty for vocal fold immobility: When is it really necessary?	2017	USA	Case-Control	Surgical – Laryngology	24	x					
