# Supplementary material for: Pharyngolaryngeal Abnormalities viewed via nasoendoscopy associated with Oropharyngeal Dysphagia in Adults: A Scoping Review
Source: Dysphagia. 2025 Sep 22;41(2):358–69. doi: 10.1007/s00455-025-10884-6 (PMC13099671; doi:10.1007/s00455-025-10884-6)
Supplement: Supplementary file 4 — Supplementary Material 4 [file 455_2025_10884_MOESM4_ESM.pdf]

Supplementary *Information 2: Inclusion-Exclusion Criteria*

Inclusions	Examples/ Variations	
Must be under endoscopic view	• Videolaryngoscop*<br>• Videoendoscop*<br>• Flexible Endoscopic Evaluation of Swallowing (FEES)<br>• Fibreoptic Endoscopic Evaluation of Swallowing (FEES)<br>• Nasoendoscop*<br>• Laryngoscop*<br>• Laryngeal video recordings<br>• Laryngologist Evaluation/ Flexible Nasendoscopy<br>• Transoral endoscopic evaluation<br>• Instrumental evaluation	
Must include endoscopic pharyngolaryngeal structures and abnormalities	• Upper airway<br>• Throat<br>• Pharynx<br>• Hypopharynx<br>○ Pharyngeal wall<br>○ Base of tongue<br>○ Epiglottis<br>○ Valleculae<br>○ Pre-epiglottic space<br>○ Lateral channels<br>○ Pyriforms/piriforms/ pyriform recess<br>• Larynx<br>○ Anterior commissure<br>○ Arytenoid<br>○ Interarytenoid/ posterior commissure<br>○ Aryepiglottic fold<br>○ Laryngeal vestibule<br>○ Vocal process<br>○ Ventricular fold<br>○ Vocal fold/cord/glottic<br>• Subglottic<br>• Laryngotracheal stenosis	• Edema / congestion<br>• Erythema / inflammation<br>• Ulceration/ Granulation / mucosal trauma<br>• Paresis /palsy /immobility/ Reduced/ restricted movement/ subluxation/ overriding/ ankylosis /dislocation<br>• Pachydermia<br>• Laryngitis<br>• Lesions<br>• Glottic web<br>• Masses<br>• Muscle tension<br>• Evidence of thermal burn to mucosa sloughy mucosa tissue<br>• Contracture<br>• Atrophy
Must relate to oropharyngeal swallowing function	• Oropharyngeal dysphagia<br>• Swallowing<br>• Timing of swallowing initiation<br>• Pharyngal phase duration<br>• Residue/ Pharyngal residue/ Pooling/ bolus retention<br>• Penetration/ laryngeal influx<br>• Spillage<br>• Sensation<br>• Aspiration<br>• Secretions / saliva stasis/ laryngeal influx	• Outcome measures<br>○ Functional Oral Intake Scale (FOIS)<br>○ AusToms<br>○ Penetration/Aspiration Scale (PAS)<br>○ New Zealand Secretion Scale (NZSS)<br>○ Yale Residue Scale<br>○ Marianjoy<br>○ International Dysphagia Diet Standardisation Initiative (IDDSI)<br>○ Dysphagia outcome and severity scale (DOSS)

	• Tracheostomy weaning (decannulation, cuff deflation)	○ Dynamic Imaging Grade of Swallowing Toxicity (DIGEST)<br>○ SWAL-QOL<br>○ VASES
**Exclusions**		
Related to oesophageal or gastrointestinal function dysfunction	• Endoscope<br>• Endoscopy<br>• Oesophageal<br>• Manometry	
Children (0-18)	• Neonatal<br>• Suckling	
Not Human		
Radiological	Videofluoroscopic/VFSS	Barium swallow<br>Modified barium swallow
Ultrasound		
Non- invasive swallowing assessment		
Interventions	Therapy Interventions/surgery with no comments related to swallowing/tracheostomy pre or post operatively	
Total Laryngectomy		
Conference abstracts with no full text available		
Literature reviews		
Foreign body ingestion where foreign body remains		
Not in English		
Nil link to change and dysphagia		
Intervention studies where abnormalities already identified and post operative swallowing function assessed – wrong study design		
