# Supplementary material for: Pharyngolaryngeal Abnormalities viewed via nasoendoscopy associated with Oropharyngeal Dysphagia in Adults: A Scoping Review
Source: Dysphagia. 2025 Sep 22;41(2):358–69. doi: 10.1007/s00455-025-10884-6 (PMC13099671; doi:10.1007/s00455-025-10884-6)
Supplement: Supplementary file 5 — Supplementary Material 5 [file 455_2025_10884_MOESM5_ESM.pdf]

Online Resource 5: Frequency counts of co-occurrence and statistical analysis

Group	Pharynolaryngeal abnormalities related to aspiration unless otherwise stated	Prevalence (CI 95%)	Meta-analysis across multiple articles or rate ratio from individual articles with aspiration (CI 95%) >1	Significant p values (p<0.05) related to aspiration unless otherwise stated	Frequency of co-occurrence – articles / participants (≥ five or ≥ 50)
**Motion impairment**	True vocal fold motion impairment – unilateral	Aspiration 41% (0.35-0.47)[27-33]<br>Residue 80% (0.65-0.90)[30]	**Meta- analysis:**<br>RR 1.49 (1.15-1.92)<br>I2 = 21%[27-30]	P = 0.04[27]<br>P = 0.036[28]<br>P = <0.001[29]<br>P = 0.012[30]<br>P = 0.001[31]<br>P = 0.002[32]<br>P = 0.0007[33]<br>P = < 0.05 with pharyngeal squeeze maneuver [1]<br>P = >0.05 Pooling, spillage and residue [30]	63 / 600
	Velopharyngeal insufficiency			P = 0.006[31]	6 / 90
	Arytenoid motion impairment			P = 0.004[27]	5 / 45
	Reduced pharyngeal wall movement				5 / 15
	True vocal fold motion impairment – bilateral not in midline				24/144
	Subluxation / ankylosis of cricoarytenoid joint				2/4
	Laterofixation of vocal fold				1/4
	Arytenoid prolapse/collapse				2/3
**Glottal insufficiency**	Incomplete glottic closure	Aspiration 63% (0.38-0.84)[27]<br>Secretion accumulation 84% (0.77-0.90)[34]	RR 2.21 (1.47-3.33)[34]	P = 0.002[27]<br>P = 0.02 secretion accumulation[34]	9 / 134
	Vocal fold atrophy/bowing			P = 0.046[49]<br>P = 0.031[50]	12/130
	Phonatory gap/chink				
**Obstruction to bolus or airflow**	Edema overall				39/891
	Edema (unspecified)	Aspiration 45% (0.35-0.54)[27]	RR 2.46 (1.53-3.96)[27]	P = <0.0001[27]	17/377
	Arytenoid/ interarytenoid edema	Aspiration 32% (0.17-0.51)[36]<br>Sensory impairment 85% (0.72-0.93)[25]	RR 1.67 (0.13-20.88)[36]	P = <0.001 sensory impairment[18]<br>P= 0.047 residue[51]	14/132
	False vocal fold edema			P = 0.02 residue[51]	1/14
	Epiglottic edema			P = 0.02 residue[51]	4/69

	Aryepiglottic edema			P = 0.001 residue[51]	1/26
	Valleculae edema			P = 0.021 residue[51]	In unspecified
	Pyriform sinus edema			P = 0.032 residue[51] P = 0.038 sensory impairment[51]	
	True vocal fold edema	Aspiration 36% (0.18-0.57) [36]	RR 2.88 (0.43-19.38)[36]		10/114
	Bilateral true vocal fold in midline position			P = < 0.00019 **no aspiration[35]**	6 / 13
	Glottic stenosis/ web, /tracheal/airway stenosis/narrowing				9 / 31
	Cricopharyngeal prominence				1/28
	Midline protrusion				4/23
**Mucosal abnormalities**	Hematoma			P = 0.021 sensory impairment[37]	6 / 47
	Ulcer/ulceration	Aspiration 9% (0.00-0.41)[36]	RR 0.22 (0.03-1.54) [36]		18 / 279
	Granulation/ granuloma	Aspiration 32% (0.13-0.57) [36]	RR 1.11 (0.38-3.19) [36]		
	Vocal fold erythema	Aspiration 32% (0.16-0.52) [36]	RR 1.61 (0.26-10.06) [36]		12 / 217
	Arytenoid erythema	Aspiration 32% (0.16-0.52) [36]	RR 0.97 (0.10-9.84) [36]		
	Unspecified lesions				8 / 210
	Sloughing				2/2
	Cyst				1/1
	Scarring/synechiae/adhesions				8/22
	Laryngeal lesion				4/5
**Change to shape of structure**	Partial removal or absence of tonsil				4 / 61
	Partial removal or absence of true vocal folds				4 / 134
	Partial removal or absence of false vocal folds				4 / 61
	Partial removal or absence of epiglottis				3 / 71
	Partial removal or absence of base of tongue				6 / 79
	Partial removal or absence of arytenoids				6/41
	Partial removal or absence of aryepiglottic folds				3/9
	Partial removal or absence of pyriform sinus and lateral walls				3/49
	Partial removal or absence of supraglottis				1/3
	Upright epiglottis/ epiglottic stump				2/36
	Supracricoid laryngectomy				4/42
**Abnormal movement pattern**	Tremulous movements of laryngopharynx				4/46
